# Phase II study of pembrolizumab (MK-3475) for relapsed/refractory classical Hodgkin Lymphoma (r/r cHL): keynote-087

**DOI:** 10.1186/2051-1426-3-S2-P146

**Published:** 2015-11-04

**Authors:** Robert Chen, Phillippe Armand, Michelle A Fanale, Vincent Ribrag, Pier Luigi Zinzani, Alejandro D Ricart, Seth Thompson, Arun Balakumaran, Daniel Molin, Margaret A Shipp, Craig H Moskowitz

**Affiliations:** 1 City of Hope Medical Center, Duarte, CA, USA; 2 Dana-Farber Cancer Institute, Boston, MA, USA; 3 The University of Texas MD Anderson Cancer Center, Houston, TX, USA; 4 Gustave Roussy Cancer Campus, Villejuif, France; 5 University of Bologna, Bologna, Italy; 6 Merck & Co., Inc., Kenilworth, NJ, USA; 7 Uppsala University, Uppsala, Sweden; 8 Memorial Sloan Kettering Cancer Center, New York, NY, USA

## Background

The prognosis is poor for patients with cHL who relapse after autologous stem-cell transplant (auto-SCT) or progress after brentuximab vedotin (BV) therapy. cHL frequently harbors genetic amplification at 9p24.1, which leads to overexpression of PD-L1 and PD-L2 on the tumor cell surface. This suggests that cHL may have a genetically determined dependence on the PD-1 pathway for survival. Pembrolizumab is a humanized monoclonal antibody against PD-1 that is designed to block the interaction of PD-1 with its ligands PD-L1 and PD-L2. Based on the frequent expression of the ligands on cHL, we sought to evaluate the safety and efficacy of pembrolizumab for R/R cHL. In a Phase I study, pembrolizumab displayed high activity in cHL, with an overall response rate of 65% and complete response rate of 20%[1]. The present Phase II study was undertaken to extend those findings in a larger cohort.

## Methods

KEYNOTE-087 (NCT02453594) is a multicenter, nonrandomized, multicohort Phase II study comprising patients with cHL who have failed to achieve a response or progressed after auto-SCT and BV (cohort 1); patients who are not eligible for auto-SCT and have failed to achieve a response or progressed after BV (cohort 2); and patients who have failed to achieve a response or progressed after auto-SCT and have not received BV after auto-SCT (cohort 3). Key eligibility criteria include age ≥18 years, measurable disease, ECOG performance status 0/1, and adequate organ function. Exclusion criteria include immunosuppression, allogeneic stem cell transplantation within 5 years, active pneumonitis, and prior anti-PD-1/PD-L1 therapy. Treatment with pembrolizumab IV 200 mg Q3W will continue for ≤24 months or until confirmed disease progression, intolerable toxicity, or physician decision. AEs are collected throughout the study and for 30 days thereafter (90 days for serious AEs). Response is centrally assessed every 12 weeks per 2007 IWG criteria. Patients who achieve a complete response can discontinue treatment and then be retreated at the time of relapse. Primary efficacy end point is objective response rate (ORR) using IWG criteria per central review secondary end points are ORR per investigator review, ORR per central review using Lugano 5-point classification, complete remission rate, duration of response, progression-free survival, and overall survival. Exploratory end points include assessments of PK profile, relationship between candidate efficacy biomarkers and antitumor activity of pembrolizumab, and efficacy in patients who continue pembrolizumab beyond documented progression. Planned enrollment is ~180 patients.

## Trial registration

ClinicalTrials.gov identifier NCT02453594.
